# Moon-like Facies by Glucocorticoid Is Associated With the Development of Diabetes and Body Image Disturbance

**DOI:** 10.1210/jendso/bvae036

**Published:** 2024-02-29

**Authors:** Takahiro Tsutsumi, Daiki Nakagomi, Kei Kobayashi, Shunichiro Hanai, Yoshiaki Kobayashi, Ryosuke Ito, Toshihisa Ishii, Hideyuki Okuma, Hiroyuki Uchinuma, Masashi Ichijo, Kyoichiro Tsuchiya

**Affiliations:** Department of Diabetes and Endocrinology, Graduate School of Interdisciplinary Research, Faculty of Medicine, University of Yamanashi, Chuo, Yamanashi 409-3898, Japan; Department of Rheumatology, Graduate School of Interdisciplinary Research, Faculty of Medicine, University of Yamanashi, Chuo, Yamanashi 409-3898, Japan; Department of Rheumatology, Graduate School of Interdisciplinary Research, Faculty of Medicine, University of Yamanashi, Chuo, Yamanashi 409-3898, Japan; Department of Rheumatology, Graduate School of Interdisciplinary Research, Faculty of Medicine, University of Yamanashi, Chuo, Yamanashi 409-3898, Japan; Department of Rheumatology, Graduate School of Interdisciplinary Research, Faculty of Medicine, University of Yamanashi, Chuo, Yamanashi 409-3898, Japan; Department of Rheumatology, Graduate School of Interdisciplinary Research, Faculty of Medicine, University of Yamanashi, Chuo, Yamanashi 409-3898, Japan; Department of Nephrology, Graduate School of Interdisciplinary Research, Faculty of Medicine, University of Yamanashi, Chuo, Yamanashi 409-3898, Japan; Department of Diabetes and Endocrinology, Graduate School of Interdisciplinary Research, Faculty of Medicine, University of Yamanashi, Chuo, Yamanashi 409-3898, Japan; Department of Diabetes and Endocrinology, Graduate School of Interdisciplinary Research, Faculty of Medicine, University of Yamanashi, Chuo, Yamanashi 409-3898, Japan; Department of Diabetes and Endocrinology, Graduate School of Interdisciplinary Research, Faculty of Medicine, University of Yamanashi, Chuo, Yamanashi 409-3898, Japan; Department of Diabetes and Endocrinology, Graduate School of Interdisciplinary Research, Faculty of Medicine, University of Yamanashi, Chuo, Yamanashi 409-3898, Japan

**Keywords:** moon-like facies, glucocorticoid therapy, glucocorticoid-induced diabetes, body image disturbance

## Abstract

**Context:**

Moon-like facies (MLF) are a typical side effect of glucocorticoid (GC) therapy; however, its predisposing factors, relationship with GC-induced complications, and effects on body image are not well understood.

**Objective:**

This study aimed to determine the predisposing factors for MLF during GC therapy; its association with GC-induced diabetes, hypertension, and dyslipidemia; and its effects on body image.

**Methods:**

This prospective observational study spanned 24 weeks and targeted patients who received GC therapy at the University of Yamanashi Hospital from June 2020 to August 2022. The MLF was defined based on the following 3 factors: (1) an increase in facial measurement lengths, (2) subjective facial changes by patients’ self-assessment using a visual analog scale; (3) objective and qualitative facial changes assessed by physicians. We examined the predisposing factors for MLF and the association of MLF with GC-induced diabetes, hypertension, dyslipidemia, and body image.

**Results:**

The cumulative incidence rate of MLF at 24 weeks was 37.6%. Predisposing factors for MLF were an initial oral prednisolone dosage of ≥ 30 mg/day [odds ratio (OR) 63.91, 95% confidence interval (CI) 5.82-701.81] and female (OR 6.66, 95% CI 1.35-32.79). MLF showed a significant association with the onset of GC-induced diabetes (OR 6.58, 95% CI 1.25-34.74). MLF was also an independent factor contributing to body image disturbance (β = −18.94, *P* = .01).

**Conclusion:**

MLF contributes to body image disturbance and is associated with the development of GC-induced diabetes; therefore, it is clinically important as a physical manifestation of GC therapy.

Moon-like facies (MLF) is a well-known side effect of glucocorticoid (GC) therapy, but very little is known about the predisposing factors for MLF and its impact on GC-induced diabetes, hypertension, and dyslipidemia. Fardet et al conducted a series of observational studies on corticosteroid-induced lipodystrophy (CIL), which manifested as MLF and/or a buffalo hump in French patients treated with GC [1-5]. They found that CIL was more likely to occur in women and young adults and was associated with increased energy intake and subsequent weight gain [2]. Additionally, CIL is reportedly associated with the onset of hyperglycemia, hypertension, and neuropsychiatric disorders [2-5]. However, no studies have focused on MLF among the complications of GC. Therefore, the factors associated with MLF and whether the onset of MLF is associated with the risk of developing GC-induced diabetes, hypertension, and dyslipidemia remain unclear.

In general, disturbances in body image due to the side effects of treatment can deteriorate the quality of life of patients [6-8] and affect their medication compliance and treatment continuation rates [8-10]. In an interview-based survey, Hale et al reported that MLF negatively affects the body image of patients with systemic lupus erythematosus and causes psychological distress, although no statistical evaluation has been conducted to date [11].

This study aimed to clarify the predisposing factors for MLF and the association of MLF with GC-induced diabetes, hypertension, dyslipidemia, and body image disturbance by performing facial measurements.

## Materials and Methods

### Participants

This study included 90 Japanese patients aged 16 years or older who were newly prescribed oral prednisolone at the Department of Rheumatology or Nephrology at the University of Yamanashi Hospital (Yamanashi, Japan), either during hospitalization or as outpatients, between June 2020 and August 2022. All included patients provided written informed consent. This study was approved by the Clinical Trial Review Board of the University of Yamanashi Hospital (Approval No. 2246). All the research methods and procedures were conducted in accordance with the ethical principles of the Declaration of Helsinki.

We excluded patients with a history of GC use within 1 year before the start of the study, including oral, intravenous, transdermal, intranasal, or intra-articular GC use. We also excluded patients who were diagnosed with generalized edema due to renal failure, heart failure, liver failure, hypoalbuminemia, or endocrine disorders by the attending physician during the observation period; those who died during the observation period; those who discontinued regular visits; those with a history of Cushing's syndrome or its comorbidities; and those who refused to participate in the study. The use of other immunosuppressive drugs was permitted.

### Study Protocol

We conducted a prospective investigation of patients who were initiated on GC therapy. Facial measurements and body image evaluations were performed weekly during hospitalization and monthly during outpatient visits. Clinical data were obtained from the medical records, including age, sex, height, body weight, blood pressure, blood glucose levels, hemoglobin A1c (HbA1c) levels, cholesterol levels, oral prednisolone dose (the daily dose at the start and the cumulative dose) during the observation period, medical history, and concurrent illnesses. The observation period was 24 weeks. If the underlying disease improved within 24 weeks and prednisolone treatment was discontinued, the time to discontinuation was included in the analysis.

### Criteria for MLF

The MLF was defined based on the following 3 factors: (1) an increase in facial measurement lengths, (2) subjective facial changes by patients’ self-assessment using a visual analog scale (VAS), and (3) objective and qualitative facial changes assessed by physicians (“Yes” or “No”). Fulfilling conditions 1 and 2; 1 and 3; or 1, 2, and 3 were defined as MLF.

**Figure 1.**
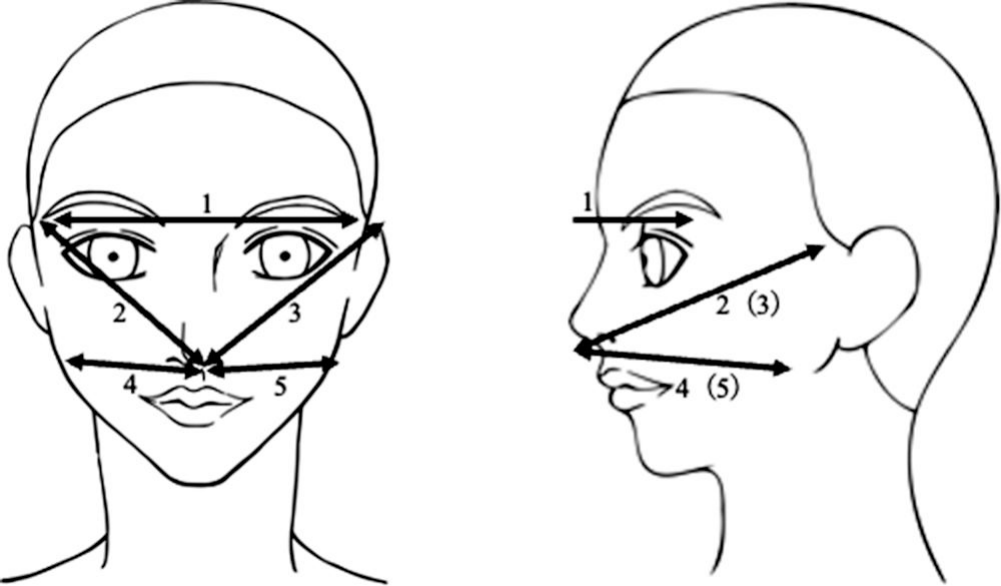
The measurement locations in this study.

The faces of the patients were measured at 5 locations by either the investigator (T.T.) or 2 specific nurses: the length between the ends of the eyebrows, the length from the nose to the upper end of the left and right auricles, and the length from under the nose to the angle of the mandible on both sides (Fig. 1). Before the start of the study, the examiners underwent multiple practice sessions to ensure consistency. To evaluate relative reliability for the facial measurement, we calculated the intraclass correlation coefficient (ICC) and its 95% confidence interval (CI) for the interrater analysis [12, 13]: the intraclass correlation coefficient (95% CI) among the 3 examiners was .99 (.96-1.00), suggesting that the internal consistency of measurement between these 3 examiners is statistically maintained (data not shown).

Measurements were taken with the same ruler and recorded in increments of .5 cm. We considered the facial measurement as significantly increased if it was increased by ≥ .5 cm after GC treatment. We used a KA-15 measure (AS ONE Co. Ltd., Japan, product #25950137) as the instrument for facial measurements as majors, which is certified by the Japanese Industrial Standards.

A VAS was used for the self-assessment of MLF by patients. As an answer to the question “How much do you think your face has changed compared to before starting the steroid treatment?”, the patients indicated their responses on the VAS consisting of a 100-mm linear scale ranging from “not changed” (0 mm) to “apparently changed” (100 mm). Fifty mm or more is defined as MLF. For the objective facial changes assessed by physicians, the physician assessed whether the patient developed MLF (“Yes” or “No”) at the same time as medical examination.

The process for evaluating and determining MLF is as follows: 1 of the 3 examiners measured the patient's face; then the patient answered a VAS before medical examination by a physician. Next, the physician subjectively assessed whether the patient developed MLF and informed the judgment to the investigator. Finally, the investigator decided whether the patient developed MLF according to the criteria. Therefore, no authority could affect the evaluation process and final decision regarding MLF development.

### Criteria for GC-induced Diabetes, Hypertension, and Dyslipidemia

GC-induced diabetes, hypertension, and dyslipidemia were evaluated after the initiation of GC therapy. GC-induced diabetes was diagnosed if the HbA1c level was 6.5% or higher, if the fasting blood glucose level was 126 mg/dL or higher, if the random blood glucose level was 200 mg/dL or higher, if the fasting blood glucose level was confirmed to be 126 mg/dL or higher on 2 separate occasions, or if medications for the treatment of diabetes (oral hypoglycemic agents, insulin, and GLP-1 receptor agonists) were started. Hypertension was diagnosed if the blood pressure at the time of examination was 140/90 mmHg or higher on 2 separate occasions or if antihypertensive medications were initiated. Dyslipidemia was diagnosed if the fasting low-density lipoprotein cholesterol level was 140 mg/dL or higher, if the high-density lipoprotein cholesterol level was less than 40 mg/dL, if the triglyceride level was 150 mg/dL or higher, or if lipid-lowering medications were initiated.

### Body Image Evaluation

To evaluate body image, the Body Image in Lupus Screen Scale (BILSv1.2), which has been shown to have positive correlations with LupusQoL, 36-item Short Form Survey, EQ5D, and BIOLI in patients with systemic lupus erythematosus [14], was used. The questionnaire was administered to the patients simultaneously with the facial measurements.

### Statistical Analysis

Statistical analyses were performed using Stata (version 17.0; Stata Corp., College Station, TX, USA). The normality of the data was confirmed using the Shapiro–Wilk test, and data that followed a normal distribution were presented as mean ± SD, while data that did not follow a normal distribution were presented as median [25th percentile value–75th percentile value]. Group comparisons were performed using the t-test, chi-squared test, or Wilcoxon rank-sum test. The cumulative incidence rates of MLF and GC-induced diabetes, hypertension, and dyslipidemia were evaluated using the Kaplan–Meier method. Factors associated with MLF were identified using multiple logistic regression analyses. Based on previous studies, the variable selection was performed using a backward selection procedure, and a *P*-value of less than .2 was used to retain the variables in the model [2]. Factors associated with GC-induced diabetes were also identified using multiple logistic regression analysis, and variables were selected based on the risk factors for GC-induced diabetes identified in previous studies [15-19]. Odds ratios (ORs) were used to evaluate the effects of the identified variables. Factors associated with scores of the BILSv1.2 were identified using linear regression analysis. Statistical significance was set at *P* < .05.

## Results

### Patient Characteristics

Among the 90 enrolled patients, 1 died due to an underlying disease, 8 refused to continue participating in the study, and 9 were excluded from the analysis because their visits to the hospital were interrupted during the observation period. A total of 72 patients were analyzed, including 69 who completed the observation period of 24 weeks, 2 who discontinued prednisolone after 20 weeks, and 1 who discontinued prednisolone after 16 weeks due to improvement in the underlying disease. Of the 72 patients, 90.3% (n = 65) started prednisolone during hospitalization.

Table 1 shows the characteristics of 72 patients. The mean initial oral prednisolone dosage was 31.8 ± 13.7 mg/day. More than half of the patients (48 of 72) received other immunosuppressive therapies.

**Table 1.** Baseline characteristics of the patients

		MLF		
	All (n = 72)	+ (n = 27)	− (n = 45)	*P*
Female, n (%)	38 (52.8)	17 (63.0)	21 (46.7)	.180
Age, years	59.7 ± 18.8	61.9 ± 15.3	58.4 ± 20.6	.460
Body weight, kg	58.5 ± 12.9	56.3 ± 10.7	59.7 ± 14.0	.284
BMI, kg/m^2^	22.5 ± 3.2	22.3 ± 2.8	22.6 ± 3.5	.638
Sum of facial measurements, cm	69.0 ± 4.0	68.3 ± 3.6	69.5 ± 4.3	.204
Initial oral prednisolone dosage, mg/day	31.8 ± 13.7	40.6 ± 8.6	26.6 ± 13.6	<.001
Pulsed steroids, n (%)	12 (16.7)	1 (3.7)	11 (24.4)	.022
Other immunosuppressive therapies, n (%)	48 (66.7)	23 (85.2)	25 (55.6)	.010
HbA1c, %	5.9 [5.5–6.4]	5.9 [5.6–6.1]	5.9 [5.5–6.8]	.767
Underlying disease				
Microscopic polyangiitis, n (%)	11 (15.3)	7 (25.9)	4 (8.9)	.052
Polymyalgia rheumatica, n (%)	7 (9.7)	2 (7.4)	5 (11.1)	.608
IgA nephropathy, n (%)	5 (6.9)	0 (0)	5 (11.1)	.073
Others	49 (68.1)	18 (66.7)	31 (68.9)	.845
Other diseases				
Diabetes, n (%)	14 (19.4)	2 (7.4)	12 (26.7)	.046
Hypertension, n (%)	27 (37.5)	8 (29.6)	19 (42.2)	.285
Dyslipidemia, n (%)	33 (45.8)	12 (44.4)	21 (46.7)	.855

Abbreviations: BMI, body mass index; HbA1c, hemoglobin A1c; MLF, moon-like facies.

Values are shown as mean ± SD and median [25th percentile value–75th percentile value].

**Figure 2.**
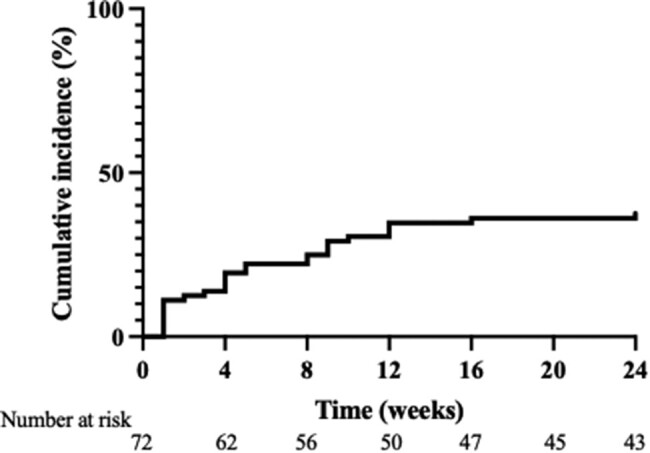
Cumulative incidence rates of moon-like facies.

A total of 27 patients developed MLF during the observation period. The cumulative incidence rates of MLF were 11.1%, 19.4%, 25.0%, and 37.6% at 1, 4, 8, and 24 weeks, respectively (Fig. 2). The numbers of patients fulfilling 1 and 2; 1 and 3; or 1, 2, and 3 of the factors in criteria for MLF, as described earlier, were 0, 21, and 6, respectively. Chi-squared test to evaluate relationships between criteria 1, 2, and 3 revealed a significant relationship between 1 and 3 (*P* = .01). In contrast, there was no significant relationship between criteria 1 and 2 (*P* = .372) (data not shown).

### Characteristics of Patients With or Without MLF

**Figure 3.**
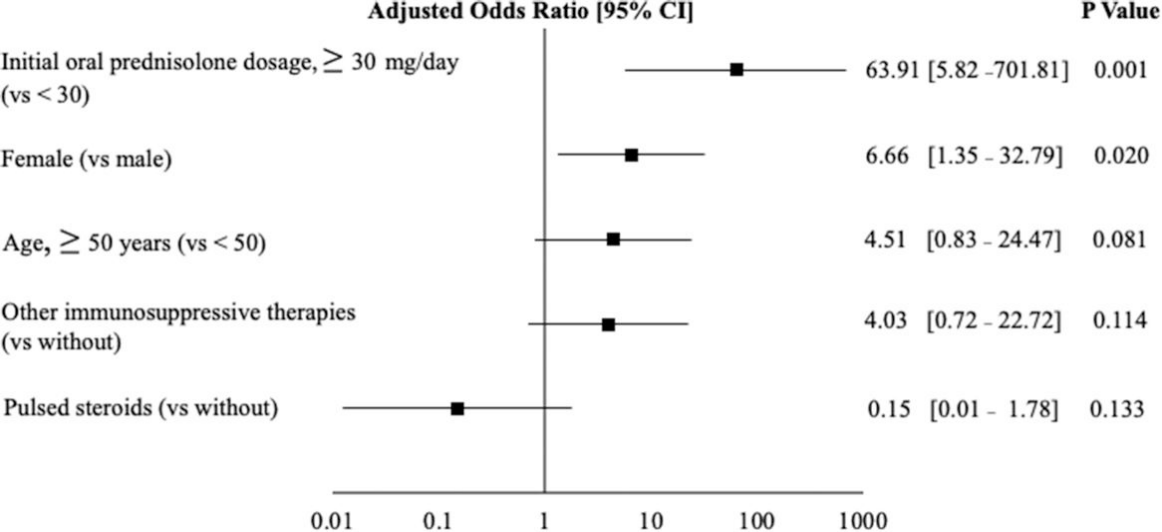
Logistic regression analysis of risk factors in patients with and without moon-like facies. Risk assessment model for moon-like facies. The black square represents the odds ratio after adjusting for these factors in the logistic regression model, and the horizontal line indicates the 95% confidence interval. Variables that showed statistical significance at the end of the logistic regression analysis are presented.

**Figure 4.**
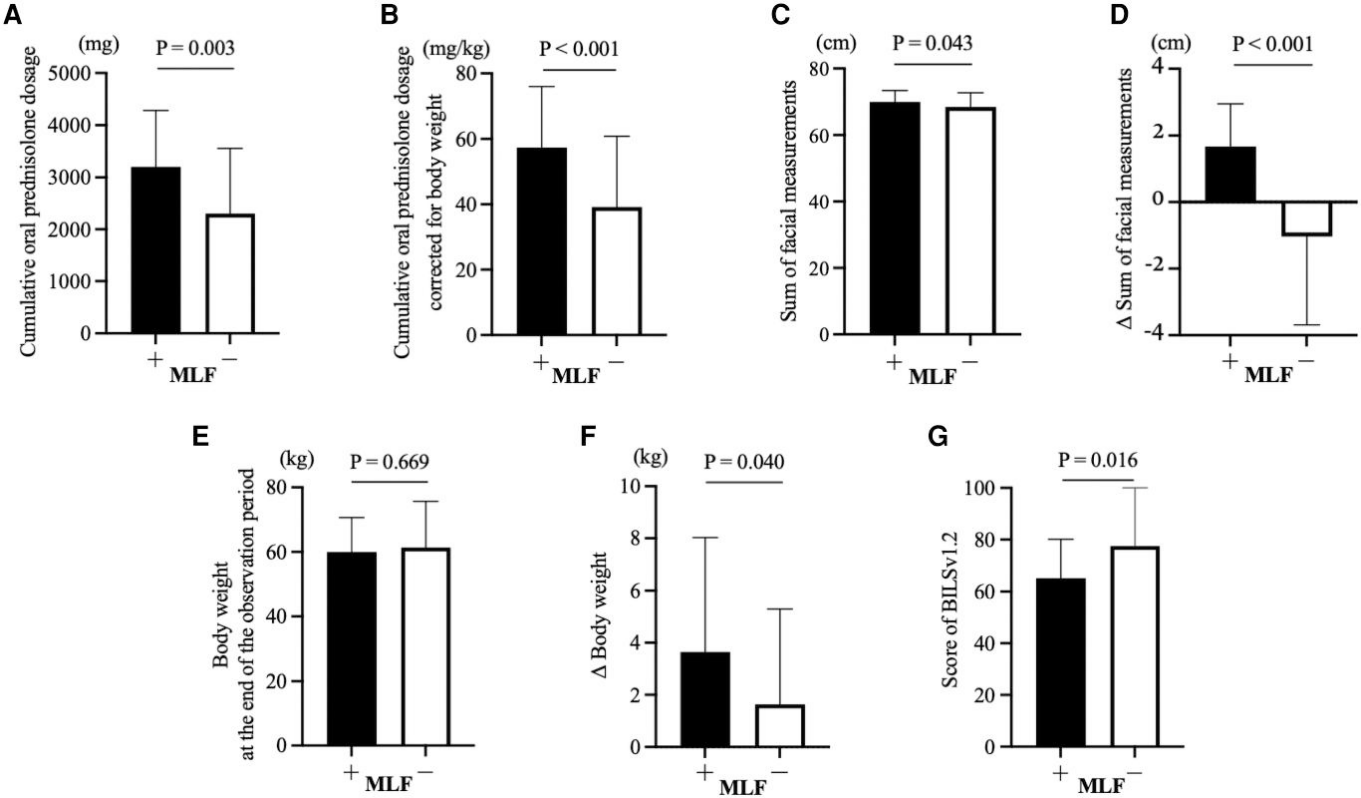
Characteristics of patients with and without MLF after glucocorticoid therapy. (A) Cumulative oral prednisolone dosage. (B) Cumulative oral prednisolone dosage corrected for body weight. (C) Sum of facial measurements. (D) Δ Sum of facial measurements. (E) Body weight at the end of the observation period. (F) Δ Body weight. (A) Score of the BILSv1.2. We excluded 10 patients who did not provide informed consent. The results were compared between the lowest scores. (C, D) Results were compared at the time of MLF determination for patients with MLF and at the end of the observation period for those without MLF. Abbreviations: BILSv1.2, Bilateral Inverted Locus of the Skin; MLF, moon-like facies.

In patients with MLF, the initial oral prednisolone dosage (40.6 ± 8.6 mg/day vs 26.6 ± 13.6 mg/day; *P* < .001) was higher compared to those without MLF (Table 1). A higher proportion of patients with MLF received other immunosuppressive therapies (85.2% vs 55.6%, *P* = .01). Patients who received pulsed steroids (3.7% vs 24.4%, *P* = .022) and those with diabetes (7.4% vs 26.7%, *P* = .046) were fewer in the MLF group. Logistic regression analysis with MLF as the dependent variable showed that the initial oral prednisolone dosage ≥30 mg/day (OR 63.91, 95% CI 5.82-701.81, *P* = .001) and female (OR 6.66, 95% CI 1.35-32.79, *P* = .02) were the significant predictors of MLF (Fig. 3). Patients with MLF were found to receive a higher cumulative oral prednisolone dosage and cumulative oral prednisolone dosage corrected for body weight than those without MLF (3200 ± 1080 mg vs 2300 ± 1260 mg, *P* = .003, 57.4 ± 18.6 mg/kg vs 39.2 ± 21.6 mg/kg, *P* < .001, respectively) (Fig. 4A and 4B). There were no significant differences in body weight at the end of the observation period based on the presence of MLF (Fig. 4E). However, the sum of facial measurements (70.2 ± 3.3 cm vs 68.3 ± 4.1 cm, *P* = .043), change in the sum of facial measurements (1.9 ± 1.3 cm vs −1.0 ± 2.8 cm, *P* < .001), and change in body weight (3.6 ± 4.4 kg vs 1.6 ± 3.7 kg, *P* = .04) were significantly increased in patients with MLF (Fig. 4C, 4D, and 4F).

### GC-induced Diabetes, Hypertension, and Dyslipidemia

**Figure 5.**
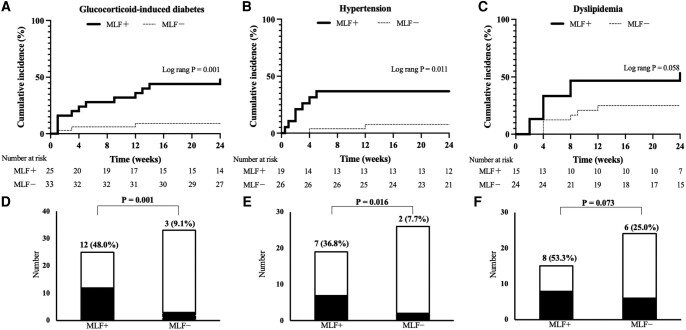
(A-C) Cumulative incidence rates of GC-induced diabetes, hypertension, and dyslipidemia. The variables with statistical significance in the log-rank test are presented. (D-F) Number of patients with GC-induced diabetes, hypertension, and dyslipidemia with or without MLF. The variables with statistical significance in the chi-square test are shown. (A, D) A total of 14 patients with diabetes at the start of the study were excluded. (B, E) A total of 27 patients with hypertension at the start of the study were excluded. (C, F) A total of 33 patients with dyslipidemia at the start of the study were excluded. Abbreviations: GC, glucocorticoid; MLF, moon-like facies.

Patients with MLF had a significantly higher incidence (48.0% vs 9.1%, *P* = .001) and cumulative occurrence rate (log-rank, *P* = .001) of GC-induced diabetes than those without MLF (Fig. 5A and 5D). Additionally, the incidence (36.8% vs 7.7%, *P* = .016) and cumulative occurrence rate (log-rank, *P* = .011) of hypertension were higher in patients with MLF (Fig. 5B and E). Although not statistically significant, the incidence of dyslipidemia tended to be higher in patients with MLF (53.3% vs 25.0%, *P* = .073) (log-rank, *P* = .058) (Fig. 5C and 5F).

Table 2 shows the characteristics of the patients who developed GC-induced diabetes. Patients who developed GC-induced diabetes had a significantly higher age (67.5 ± 17.0 years vs 52.8 ± 18.6 years, *P* = .009), HbA1c levels (6.0 ± .42% vs 5.7 ± 0.41%, *P* = .016), and cumulative oral prednisolone dosage (57.5 [44.5-67.6] mg/kg vs 43.3 [30.5-60.6] mg/kg, *P* = .046) compared to those who did not develop this condition. MLF was also more frequent in patients who developed GC-induced diabetes compared to those who did not (80.0% vs 30.2%, *P* = .001). Additionally, patients who developed GC-induced diabetes showed a substantial increase in facial measurements (80.0% vs 46.5%, *P* = .025), particularly within the first month of treatment (53.3% vs 14.0%, *P* = .002). These findings suggest that an initial increase in facial measurements was associated with the onset of GC-induced diabetes.

**Table 2.** Characteristics of patients with GC-induced diabetes

	GC-induced diabetes		
	+ (n = 15)	− (n = 43)	*P*
Female, n (%)	10 (66.7)	24 (55.8)	.462
Age, years	67.5 ± 17.0	52.8 ± 18.6	.009
MLF, n (%)	12 (80.0)	13 (30.2)	.001
Increase in facial measurements, n (%)	12 (80.0)	20 (46.5)	.025
Increase in facial measurements in the first month, n (%)	8 (53.3)	6 (14.0)	.002
HbA1c before prednisolone treatment, %	6.0 ± 0.42	5.7 ± 0.41	.016
Initial oral prednisolone dosage, mg/day	37.0 ± 13.3	32.2 ± 13.3	.235
Cumulative oral prednisolone dosage, mg/kg	57.5 [44.5–67.6]	43.3 [30.5–60.6]	.046
BMI before prednisolone treatment, kg/m^2^	21.9 ± 2.6	21.9 ± 3.0	.895
Δ Body weight, kg	2.1 ± 3.6	3.3 ± 4.0	.320
No weight gain, n (%)	6 (40.0)	8 (18.6)	.095
Increased facial measurements and no weight gain, n (%)	6 (40.0)	1 (2.3)	<.001
Pulsed steroids, n (%)	2 (13.3)	9 (20.9)	.518
Other immunosuppressive therapies, n (%)	12 (80.0)	28 (65.1)	.283

Abbreviations: BMI, body mass index; GC, glucocorticoid; HbA1c, hemoglobin A1c; MLF, moon-like facies.

We excluded 14 patients with diabetes at the start of the study. Values are shown as mean ± SD and median [25th percentile value–75th percentile value].

**Figure 6.**
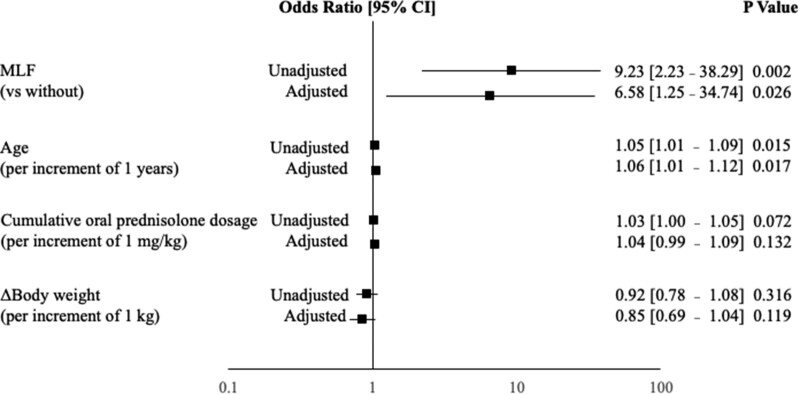
Logistic regression analysis of risk factors with GC-induced diabetes as the dependent variable. Risk assessment model for GC-induced diabetes. The black square represents the odds ratio in the univariate and multivariate analysis, and the horizontal line indicates the 95% confidence interval. Variables that showed statistical significance at the end of the logistic regression analysis are presented. Abbreviation: GC, glucocorticoid.

Figure 6 shows the results of the logistic regression analysis with GC-induced diabetes as the dependent variable and MLF and the risk factors previously reported for GC-induced diabetes as the independent variables [15-19]. In univariate analysis, MLF (OR 9.23, 95% CI 2.23-38.29, *P* = .002) and age (OR 1.05, 95% CI 1.01-1.09, *P* = .015) were found to be the significant factors. In multivariate analysis, MLF (OR 6.58, 95% CI 1.25-34.74, *P* = .026) and age were still significant (OR 1.06, 95% CI 1.01-1.12, *P* = .017), and MLF was found to be an independent factor associated with GC-induced diabetes.

In 8 (66.7%) out of 12 patients who had both MLF and GC-induced diabetes, MLF preceded or occurred simultaneously with GC-induced diabetes. Additionally, the average onset time for GC-induced diabetes was 7.3 ± 6.9 weeks, while it was 4.9 ± 4.6 weeks for MLF, indicating that MLF preceded the onset of GC-induced diabetes (data not shown).

### Body Image Evaluation

**Figure 7.**
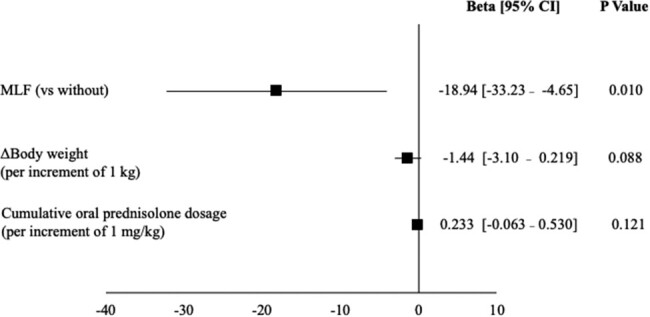
Linear regression analysis with scores of Bilateral Inverted Locus of the Skin as the dependent variable. The black square represents the β coefficient after adjusting for these factors in the linear regression analysis, and the horizontal line indicates the 95% confidence interval. Variables that showed statistical significance at the end of the logistic regression analysis are presented. Ten patients who did not provide informed consent were excluded.

Patients with MLF had significantly lower scores in the BILSv1.2 compared to those without MLF, as shown in Fig. 4G (65.0 [40.0-80.0] vs 77.5 [70.0-100], *P* = .016). In the multivariate analysis with the BILSv1.2 as the dependent variable, MLF was selected (β = −18.94, 95% CI −33.23–−4.65, *P* = .01) as an independent factor contributing to body image disturbance (Fig. 7). These results demonstrate that MLF is an independent risk factor for body image disturbance.

## Discussion

The present study shows that the onset of MLF during GC treatment is associated with the development of GC-induced diabetes and hypertension. Notably, the onset of MLF was found to be an independent factor associated with GC-induced diabetes.

In previous studies on high-dose prednisolone administration (≥ 20 mg/day), CIL was found to be associated with hyperglycemia, hypertension, and cardiovascular events [3]. Our study uses a novel approach as we distinguished MLF from CIL and included patients treated with a broad range of prednisolone doses (10-50 mg/day) compared to those in previous studies [1-5].

There has been no report showing biometric or quantitative assessment for MLF. A previous report has characterized facial features of healthy subjects with high salivary cortisol levels using computer image analysis [20]: high salivary cortisol levels are associated with facial features such as a broadened perimeter of the face, a rounded facial contour, and swollen, slit-like eyes with increased extracellular plasma volume. In the present study, locations 2, 3, 4, and 5 can reflect a broadened perimeter of the face and a rounded facial contour, and location 1 can reflect swollen, slit-like eyes. Therefore, we considered these measurement locations in the present study to reflect the facial features of GC-associated facial changes.

The pathophysiology of MLF and central obesity is not completely clear, partly due to the lack of available animal models [21, 22]. However, the following mechanisms have been proposed for GC-induced central obesity: The glucocorticoid receptor (GR) is more abundant in visceral adipose tissue than in subcutaneous adipose tissue [23, 24]. Therefore, GC may have a greater effect on lipoprotein lipase activity in visceral adipose tissue, resulting in visceral fat accumulation, because GR promotes lipoprotein lipase activity [24-26]. Additionally, GC promotes ectopic fat accumulation. Ectopic lipid accumulation in the liver and muscles caused by GC causes insulin resistance and promotes hyperglycemia [19].

In this study, an initial oral prednisolone dosage ≥30 mg/day and female sex were identified as the predisposing factors for MLF. This is consistent with the findings of previous studies in which GC dosage was correlated with the incidence of GC-induced diabetes [15-19]. GC regulates glucose intolerance and adiposity. Therefore, during the pathogenesis of GC-induced diabetes, it is thought that GC dose-dependently increases the expression of glycogenic genes via the GR, such as phosphoenolpyruvate carboxykinase and glucose-6-phosphatase [19]. Furthermore, the GR reportedly upregulates gene expression of fatty acid synthase and acetyl-CoA carboxylase, which stimulate adipogenesis [22]. Thus, we hypothesized that dose-dependent facial adipogenesis induced by GC is involved in the pathogenesis of MLF. Similar to the present study, previous studies have shown that female sex is a predisposing factor for CIL [2], although the underlying mechanism has not yet been elucidated. lipoprotein lipase activity in human subcutaneous fat is known to be higher in females than in males [27]. Therefore, this may promote the accumulation of facial subcutaneous fat in females.

Patients who developed GC-induced diabetes had significantly increased facial measurements but did not experience weight gain. These patients were older than those without GC-induced diabetes. We speculate that they gained fat instead of losing muscle. Previous studies have shown that male hormones inhibit GC-induced skeletal muscle atrophy [28] and that women are more susceptible to muscle weakness in subclinical hypercortisolism [29]. Additionally, GC-induced skeletal muscle atrophy is more likely to advance in female mice than in males [30]. Although our study did not specifically evaluate the muscles, the loss of muscle mass may play a role in the pathogenesis of GC-induced diabetes.

In this study, patients who developed MLF experienced body image disturbances. Furthermore, patients with MLF exhibited a significant increase in body weight compared to those without MLF. In a previous study, the main reason why patients with rheumatoid arthritis refused GC therapy was an increase in body weight [31, 32]. In multivariate analysis adjusted for body weight, MLF was found to be an independent factor for body image disturbance. Therefore, healthcare professionals should provide psychological support to patients with MLF and explain the potential development of MLF in patients with risk factors before initiating treatment.

The limitations of this study include its small sample size, single-center design, and being restricted to Asian individuals. Furthermore, facial measurement as an evaluation method for the MLF is a new approach, and the optimal facial measurement locations for assessing the MLF have not yet been established. Therefore, it cannot be claimed that the 5 facial areas measured in this study were optimal for evaluating MLF.

In conclusion, we identified the predisposing factors for MLF and its association with GC-induced diabetes and body image disturbance by utilizing a combination of facial measurements and subjective assessments by individuals and physicians. However, further research is needed to elucidate the pathophysiology of MLF and determine the appropriate facial measurement methods for its objective assessment.

## Data Availability

Some or all datasets generated during and/or analyzed during the current study are not publicly available but are available from the corresponding author on reasonable request.

## References

[1] Fardet L, Flahault A, Kettaneh A, et al Corticosteroid-induced clinical adverse events: frequency, risk factors and patient's opinion. Br J Dermatol. 2007;157(1):142‐148. 17501951 10.1111/j.1365-2133.2007.07950.x

[2] Fardet L, Cabane J, Lebbé C, et al Incidence and risk factors for corticosteroid-induced lipodystrophy: a prospective study. J Am Acad Dermatol. 2007;57(4):604‐609. 17582650 10.1016/j.jaad.2007.04.018

[3] Fardet L, Cabane J, Kettaneh A, et al Corticosteroid-induced lipodystrophy is associated with features of the metabolic syndrome. Rheumatology (Oxford). 2007;46(7):1102‐1106. 17409127 10.1093/rheumatology/kem062

[4] Fardet L, Kettaneh A, Tiev KP, et al Digital photography as an operational tool for assessing corticosteroid-induced lipodystrophy. Eur J Intern Med. 2008;19(5):340‐344. 18549936 10.1016/j.ejim.2007.09.015

[5] Fardet L, Flahault A, Kettaneh A, et al Corticosteroid-induced clinical adverse events: frequency, risk factors and patient's opinion. Br J Dermatol. 2007;157(1):142‐148. 17501951 10.1111/j.1365-2133.2007.07950.x

[6] Rosenberg SM, Dominici LS, Gelber S, et al Association of breast cancer surgery with quality of life and psychosocial well-being in young breast cancer survivors. JAMA Surg. 2020;155(11):1035‐1042. 32936216 10.1001/jamasurg.2020.3325 PMC7495332

[7] Albert JG, Lo C, Rosberger Z, et al Biopsychosocial markers of body image concerns in patients with head and neck cancer: a prospective longitudinal study. Curr Oncol. 2022;29(7):4438‐4454. 35877213 10.3390/curroncol29070353 PMC9319375

[8] Sloan M, Lever E, Gordon C, et al Medication decision-making and adherence in lupus: patient-physician discordance and the impact of previous “adverse medical experiences”. Rheumatology (Oxford). 2022;61(4):1417‐1429. 34247235 10.1093/rheumatology/keab534 PMC8996780

[9] Sun K, Coles TM, Voils CI, et al Development and initial validation of a systemic lupus erythematosus-specific measure of the extent of and reasons for medication nonadherence. J Rheumatol. 2022;49(12):1341‐1348. 36243406 10.3899/jrheum.220399 PMC9722566

[10] Treharne GJ, Lyons AC, Hale ED, et al Compliance” is futile but is “concordance” between rheumatology patients and health professionals attainable? Rheumatology (Oxford). 2006;45(1):1‐5. 16361701 10.1093/rheumatology/kei223

[11] Hale ED, Radvanski DC, Hassett AL. The man-in-the-moon face: a qualitative study of body image, self-image and medication use in systemic lupus erythematosus. Rheumatology (Oxford). 2015;54(7):1220‐1225. 25550393 10.1093/rheumatology/keu448

[12] Koo TK, Li MY. A guideline of selecting and reporting intraclass correlation coefficients for reliability research. J Chiropr Med. 2016;15(2):155‐163. 27330520 10.1016/j.jcm.2016.02.012 PMC4913118

[13] Bruton A, Conway JH, Holgate ST. Reliability: what is it, and how is it measured? Physiotherapy. 2000;86(2):94‐99.

[14] Jolly M, Pickard AS, Sequeira W, et al A brief assessment tool for body image in systemic lupus erythematosus. Body Image. 2012;9(2):279‐284. 22154813 10.1016/j.bodyim.2011.11.001

[15] Wu J, MacKie SL, Pujades-Rodriguez M. Glucocorticoid dose-dependent risk of type 2 diabetes in six immune-mediated inflammatory diseases: a population-based cohort analysis. White rose research online 2020. BMJ Open Diabetes Res Care. 2020;8(1):e001220. 10.1136/bmjdrc-2020-001220 PMC7389515 32719077

[16] Gonzalez-Gonzalez JG, Mireles-Zavala LG, Rodriguez-Gutierrez R, et al Hyperglycemia related to high-dose glucocorticoid use in noncritically ill patients. Diabetol Metab Syndr. 2013;5(1):18. 23557386 10.1186/1758-5996-5-18 PMC3635995

[17] Hwang JL, Weiss RE. Steroid-induced diabetes: a clinical and molecular approach to understanding and treatment. Diabetes Metab Res Rev. 2014;30(2):96‐102. 24123849 10.1002/dmrr.2486 PMC4112077

[18] Nowak KM, Rdzanek-Pikus M, Romanowska-Próchnicka K, Nowakowska-Płaza A, Papierska L. High prevalence of steroid-induced glucose intolerance with normal fasting glycaemia during low-dose glucocorticoid therapy: an oral glucose tolerance test screening study. Rheumatology (Oxford). 2021;60(6):2842‐2851. 33254223 10.1093/rheumatology/keaa724

[19] Li J-X, Cummins CL. Fresh insights into glucocorticoid-induced diabetes mellitus and new therapeutic directions. Nat Rev Endocrinol. 2022;18(9):540‐557. 35585199 10.1038/s41574-022-00683-6 PMC9116713

[20] Windhager S, Bookstein FL, Millesi E, Wallner B, Schaefer K. Patterns of correlation of facial shape with physiological measurements are more integrated than patterns of correlation with ratings. Sci Rep. 2017;7(1):45340. 28349947 10.1038/srep45340 PMC5368612

[21] Wang J-C, Gray NE, Kuo T, Harris CA. Regulation of triglyceride metabolism by glucocorticoid receptor. Cell Biosci. 2012;2(1):19. 22640645 10.1186/2045-3701-2-19 PMC3419133

[22] Lee M-J, Pramyothin P, Karastergiou K, et al Deconstructing the roles of glucocorticoids in adipose tissue biology and the development of central obesity. Biochim Biophys Acta. 2014;1842(3):473‐481. 23735216 10.1016/j.bbadis.2013.05.029 PMC3959161

[23] Brönnegård M, Arner P, Hellström L, Akner G, Gustafsson J-Å. Glucocorticoid receptor messenger ribonucleic acid in different regions of human adipose tissue. Endocrinology. 1990;127(4):1689‐1696. 2401232 10.1210/endo-127-4-1689

[24] Pedersen SB, Jønler M, Richelsen B. Characterization of regional and gender differences in glucocorticoid receptors and lipoprotein lipase activity in human adipose tissue. J Clin Endocrinol Metab. 1994;78(6):1354‐1359. 8200937 10.1210/jcem.78.6.8200937

[25] Gathercole LL, Morgan SA, Bujalska IJ, Hauton D, Stewart PM, Tomlinson JW. Regulation of lipogenesis by glucocorticoids and insulin in human adipose tissue. PLoS One. 2011;6(10):e26223. 22022575 10.1371/journal.pone.0026223 PMC3194822

[26] Panarotto D, Poisson J, Devroede G, Maheux P. Lipoprotein lipase steady-state mRNA levels are lower in human omental versus subcutaneous abdominal adipose tissue. Metab Clin Exp. 2000;49(9):1224‐1227. 11016909 10.1053/meta.2000.8624

[27] Votruba SB, Jensen MD. Sex differences in abdominal, gluteal, and thigh LPL activity. Am J Physiol Endocrinol Metab. 2007;292(6):E1823‐E1828. 17311894 10.1152/ajpendo.00601.2006

[28] Crawford BAL, Liu PY, Kean MT, Bleasel JF, Handelsman DJ. Randomized placebo-controlled trial of androgen effects on muscle and bone in men requiring long-term systemic glucocorticoid treatment. J Clin Endocrinol Metab. 2003;88(7):3167‐3176. 12843161 10.1210/jc.2002-021827

[29] Kim JH, Kwak MK, Ahn SH, et al Alteration in skeletal muscle mass in women with subclinical hypercortisolism. Endocrine. 2018;61(1):134‐143. 29717464 10.1007/s12020-018-1598-0

[30] Li S, Schönke M, Buurstede JC, et al Sexual dimorphism in transcriptional and functional glucocorticoid effects on mouse skeletal muscle. Front Endocrinol (Lausanne). 2022;13:907908. 35898460 10.3389/fendo.2022.907908 PMC9309696

[31] Morrison E, Crosbie D, Capell HA. Attitude of rheumatoid arthritis patients to treatment with oral corticosteroids. Rheumatology (Oxford). 2003;42(10):1247‐1250. 12832705 10.1093/rheumatology/keg355

[32] Nassar K, Janani S, Roux C. Long-term systemic glucocorticoid therapy: patients’ representations, prescribers’ perceptions, and treatment adherence. Joint Bone Spine. 2014;81(1):64‐68. 23953225 10.1016/j.jbspin.2013.07.001

