# Correction to: “Moon-like Facies by Glucocorticoid Is Associated With the Development of Diabetes and Body Image Disturbance”

**DOI:** 10.1210/jendso/bvae077

**Published:** 2024-04-22

In the above-named article by Tsutsumi T, Nakagomi D, Kobayashi K, Hanai S, Kobayashi Y, Ito R, Ishii T, Okuma H, Uchinuma H, Ichijo M, and Tsuchiya K (*J Endo Society*. 2024; 8(5); doi: 10.1210/jendso/bvae036), there was an error in the Materials and Methods section.

In the originally published article, in the Materials and Methods section, under the “Body Image Evaluation” heading, the first sentence read: “To evaluate body image, the Bilateral Inverted Locus of the Skin questionnaire (BILSv1.2), which has been shown to have positive correlations with LupusQoL, 36-item Short Form Survey, EQ5D, and BIOLI in patients with systemic lupus erythematosus, was used.” In the corrected article, “Bilateral Inverted Locus of the Skin questionnaire” was changed to “Body Image in Lupus Screen Scale” and the sentence now reads: “To evaluate body image, the Body Image in Lupus Screen Scale (BILSv1.2), which has been shown to have positive correlations with LupusQoL, 36-item Short Form Survey, EQ5D, and BIOLI in patients with systemic lupus erythematosus, was used.”

The article has been corrected online.

doi: 10.1210/jendso/bvae036

